# Supplementary material for: Discovery of Bis-Imidazoline Derivatives as New CXCR4 Ligands
Source: Molecules. 2023 Jan 24;28(3):1156. doi: 10.3390/molecules28031156 (PMC9920567; doi:10.3390/molecules28031156)
Supplement: Supplementary file 1 [file molecules-28-01156-s001.zip › molecules-2144479-supplementary.pdf]

Figure S1

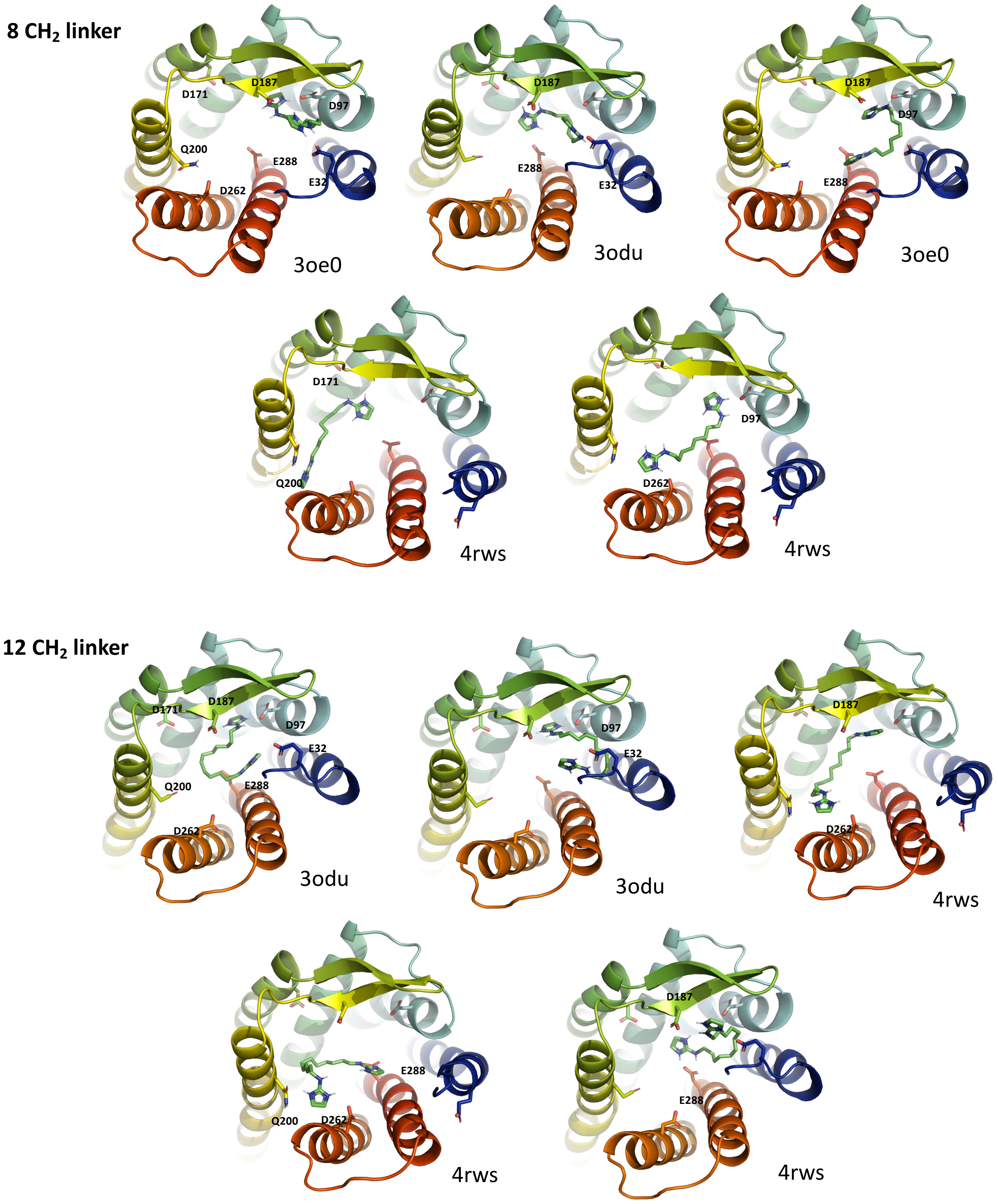

Figure S1: Proposed models of the binding mode of bis-imidazoline compounds mr20347 and mr20350 to CXCR4 from docking studies. The receptor is in ribbon and bis-imidazoline compounds are in stick representation in green. This figure was made with PYMOL.
